# Efficacy of nasal irrigation with hypertonic saline on chronic rhinosinusitis: systematic review and meta-analysis^☆^

**DOI:** 10.1016/j.bjorl.2020.03.008

**Published:** 2020-05-16

**Authors:** Lei Liu, Min Pan, Yimin Li, Guojing Tan, Yucheng Yang

**Affiliations:** Chongqing Medical University, The First Affiliated Hospital, Department of Otorhinolaryngology, Chongqing, China

**Keywords:** Chronic rhinosinusitis, Nasal irrigation, Hypertonic saline, Treatment, Meta-analysis, Rinossinusite crônica, Irrigação nasal, Solução salina hipertônica, Tratamento, Metanálise

## Abstract

**Introduction:**

Currently, several different concentrations of saline are recommended for use in nasal irrigation. Increasing studies show that nasal irrigation with hypertonic saline is more effective than traditional saline in the treatment of rhinosinusitis, but there have been few systematic analyses of the effect of nasal irrigation with hypertonic saline on chronic rhinosinusitis.

**Objective:**

We sought to compare the effects of hypertonic saline and isotonic saline in the treatment of rhinosinusitis in order to provide a reference for clinical nasal irrigation for chronic rhinosinusitis treatment.

**Methods:**

Medline, cochrane library, EMBASE, PubMed, Chinese biomedical journal database, China national knowledge infrastructure, Wanfang database, and other databases were searched, and the searching was supplemented by manual searches for relevant references to treatment of rhinosinusitis by saline nasal irrigation. The last retrieval date was March 2018. The included studies were evaluated for quality, and data were extracted for meta-analysis using RevMan 5.3.

**Results:**

Seven studies were included. Effects favoring hypertonic saline on nasal symptoms were greater in 4 subgroups. These were (1) patients with nasal secretion (SMD = 1.52; 95% CI: 1.04, 2.00; *p* < 0.01), (2) patients with congestion (SMD = 1.52; 95% CI: 1.04, 2.00; *p* < 0.01), (3) patients with headache (SMD = 0.82; 95% CI: 0.38, 1.26; *p* < 0.01), (4) patients with overall symptomatic relief (SMD = 1.63; 95% CI: 0.83, 2.44; *p* < 0.01). However, no difference was shown in smell improvement (SMD = 0.47; 95% CI: −0.65, 1.59; *p* = 0.41) and radiologic scores improvement (SMD = 2.44; 95% CI: -3.14, 8.02; *p* < 0.01). Besides, hypertonic saline showed greater improvement in mucociliary clearance time scores than did the isotonic saline group (SMD = 1.19; 95% CI: 0.78, 1.60; *p* < 0.01). Hypertonic saline brought greater minor adverse effects.

**Conclusion:**

Compared with isotonic saline, hypertonic saline nasal irrigation for the treatment of chronic rhinosinusitis is significantly more effective and has mild side effects in improving nasal symptoms and ciliary movement, but there is no significant difference in imaging findings and smell improvement. Although hypertonic saline is worthy of widespread use in clinical practice, it is still necessary to further study the exact manner and concentration of nasal irrigation.

## Resumo

**Introdução:**

Atualmente, o uso de várias concentrações diferentes de solução salina é recomendado na irrigação nasal. Um número crescente de estudos mostra que a irrigação nasal com solução salina hipertônica é mais eficaz do que a solução salina tradicional no tratamento de rinossinusite, mas existem poucas análises sistemáticas do efeito da irrigação nasal com solução salina hipertônica em rinossinusite crônica.

**Objetivo:**

Comparar os efeitos da solução salina hipertônica com a solução salina isotônica no tratamento da rinossinusite, para fornecer uma referência clínica de irrigação nasal no tratamento da rinossinusite crônica.

**Método:**

Foram pesquisados os bancos de dados Medline, Cochrane Library, Embase, PubMed, *Chinese biomedical journal database*, *China national knowledge infrastructure*, *Wanfang database* e outros, e a pesquisa foi complementada por pesquisas manuais de referências relevantes ao tratamento da rinossinusite por irrigação nasal com solução salina. A data da última recuperação de dados foi março de 2018. Os estudos incluídos foram avaliados quanto à qualidade e os dados foram extraídos para a metanálise com o software RevMan 5.3.

**Resultados:**

Sete estudos foram incluídos. Os efeitos favoráveis à solução salina hipertônica nos sintomas nasais foram maiores em quatro subgrupos. Esses foram (1) pacientes com secreção nasal (DMP = 1,52; IC95%: 1,04, 2,00; *p* < 0,01); (2) pacientes com congestão (DMP = 1,52; IC95%: 1,04, 2,00; *p* < 0,01); (3) Pacientes com dor de cabeça (DMP = 0,82; IC95%: 0,38, 1,26; *p* < 0,01); (4) Pacientes com alívio sintomático geral (DMP = 1,63; IC95%: 0,83, 2,44; *p* < 0,01). Entretanto, não houve diferença na melhoria do olfato (DMP = 0,47; IC95%: −0,65, 1,59; *p* = 0,41) e na melhoria dos escores radiológicos (DMP = 2,44; IC95%: −3,14, 8,02; *p* < 0,01). Além disso, o grupo solução salina hipertônica mostrou maior melhora nos escores do tempo de *clearence* mucociliar do que o grupo solução salina isotônica (DMP = 1,19; IC95%: 0,78, 1,60; *p* < 0,01). A solução salina hipertônica causou efeitos adversos menores.

**Conclusão:**

Em comparação com a solução salina isotônica, a irrigação nasal com solução salina hipertônica para o tratamento da rinossinusite crônica é significantemente mais eficaz e apresenta efeitos colaterais mais leves, melhoria mais acentuada dos sintomas nasais e no movimento ciliar, mas não há diferença significante nos achados de imagem e na melhoria do olfato. Embora a solução salina hipertônica seja digna de uso generalizado na prática clínica, ainda são necessários mais estudos sobre a forma de uso e a concentração ideal da solução nasal.

## Introduction

Chronic rhinosinusitis (CRS), defined as a condition of inflammation in the paranasal sinus mucosa persisting for more than 12 weeks, is a common disease worldwide, with a prevalence between 6% and 27.1%.^1^, ^2^, ^3^, ^4^, ^5^ CRS is associated with a significantly impaired quality of life^6^ and accounts for substantial health burdens.^5^, ^6^, ^7^ Therefore, the application and popularization of a simple and effective therapeutic regimen are in great demand.

Nasal irrigation is a common auxiliary treatment method, regarded as a simple and effective adjunct in the treatment of a variety of sinonasal disease, which is recommended by the UCSD (University of California, San Diego) nasal dysfunction clinic and otorhinolaryngologists worldwide.^8^, ^9^, ^10^, ^11^ When saline nasal irrigation washes out secretions and antigens, it physiologically propels a superficial gel layer, increases hydration in the sol layer, and enhances mucociliary function. In addition, saline nasal irrigation removes inflammatory mediators, thus resulting in better control of adverse nasal symptoms.^12^ Consequently, nasal irrigation exerts its effect not only in the relief of nasal symptoms but also in restrains inflammation and accordingly has been recommended as an adjunctive treatment for rhinosinusitis, allergic rhinitis and other sinonasal diseases.^13^

Recently, more otolaryngologists noticed that hypertonic saline was more effective than isotonic saline in nasal irrigation. Hypertonic solution, with higher osmotic pressure, allows for higher efficacy in reducing mucosal edema. Although there have been some prospective studies on the efficacy of different saline concentrations in the treatment of CRS, the clinical effectiveness of nasal irrigation with hypertonic saline remains unclear, and reasonable clinical recommendations cannot be made because of the lack of a systematic evaluation of its effectiveness. In order to investigate the evidence for efficacy and safety of hypertonic saline in the clinical management of CRS, we performed this systematic review and meta-analysis, including randomized controlled trials where patients suffer from CRS and were treated with hypertonic saline nasal irrigation to provide more reliable clinical evidence.

## Methods

### Eligibility criteria

#### Type of study

Published randomized controlled trials or quasi-randomized controlled trials of CRS treated with hypertonic saline were included.

#### Participants

The participants included adults who were clinically diagnosed with CRS. The following patients were excluded: (1) patients with functional nasal surgery, (2) patients with acute upper respiratory tract infections, (3) patients with acute rhinosinusitis, (4) patients with clinically severe metabolic, cardiovascular, immune, neurological, hematological, gastrointestinal, cerebrovascular, respiratory diseases or anything that the clinicians considered might interfere with the assessment of the results of the study or affect the safety of the subject.

#### Intervention

Studies assessing the effects of hypertonic saline compared with isotonic saline were included. Any delivery method, saline concentration, frequency, and duration of saline treatment were included.

### Outcomes

Studies were included when they assessed the following outcomes: nose symptom score (visual analog pain scale), mucociliary clearance time (saccharin clearing time) and imaging scores.

### Information sources and search strategy

Electronic searches were conducted in Medline, Cochrane Library, EMBASE, PubMed, Chinese Biomedical Journal Database, China National Knowledge Infrastructure, Wanfang Database and other databases, and were supplemented by manual searches. The date of the searches was March 2018. A combination of MESH terms and keywords was used as follows: “hypertonic solution”, “isotonic solutions”, “saline solutions”, “sodium chloride”, “nose disease”, “chronic disease”, “paranasal disease”, “chronic rhinitis”, “chronic sinusitis”, “chronic rhinosinusitis”, “nasal irrigation”, “nasal spray” and “treatment”.

### Study records: data management, selection process and data collection process

First, the appraisers read the title of the article, and then read the relevant literature abstracts and selected documents that initially met the inclusion criteria; they then read the full text. The two appraisers independently applied the exclusion criteria for document screening and classified the documents that met the inclusion criteria. The appraisers used Kappa values to calculate the consistency of the assessment, and if there was a disagreement, it was ultimately resolved by discussion.

### Risk of bias in individual studies

The quality of included studies was assessed by evaluating the risk of bias according to the Cochrane Handbook for Systematic Reviews of Interventions. Six domains were assessed: random sequence generation, allocation concealment, blinding of participants, blinding of outcome assessment, incomplete outcome data and selective reporting. The included studies had a low risk of bias when the methods used for each domain were clearly described. They had a high risk of bias when high risk was shown under the description. Unclear risk of bias was determined when there was insufficient information to judge.

### Data synthesis

Data were pooled for meta-analysis. Treatment effects of all continuous outcomes were presented as Standardized Mean Difference (SMD) with Standard Deviation (SD) and 95% Confidence Interval (CI). When total symptom score improvement was missing, the sum of individual score improvement was used for analysis. When the data included in each study were sufficiently similar (*p *≥ 0.10, *I*^2^ ≤ 50%), a fixed effect model was used for the combined analysis; if the studies had clinical homogeneity with significant heterogeneity, then a random effect model was used for the combined analysis. Statistical assessments were performed using Review Manager (RevMan) version 5.3 (The Nordic Cochrane Centre, The Cochrane Collaboration, Copenhagen, Denmark), and the significance of discrepancies in estimates of treatment effects from different trials was assessed by Cochran's *Q* test for heterogeneity and by measurement of the *I*^2^ statistic. An *I*^2^ of less than 40%, 40%–60% and greater than 60% represented low, moderate and substantial heterogeneity, respectively. Otherwise sensitivity analysis was performed to test whether the effects were still significant. To incorporate crossover trials in a meta-analysis, all measurements from both hypertonic and isotonic periods were analyzed as if the trials were performed in parallel.

## Results

### Study selection

**Figure 1:**
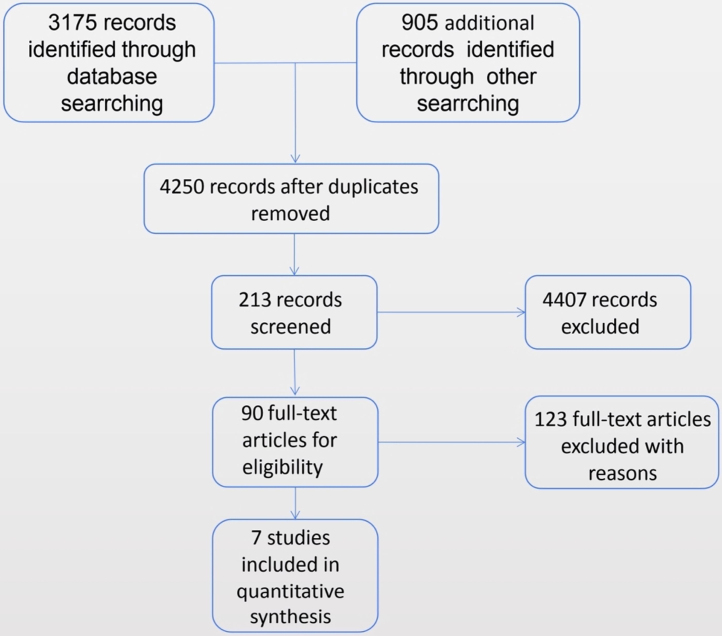
Flow chart of study retrieval and selection.

A total of seven randomized controlled trials meet our inclusion criteria.^14^, ^15^, ^16^, ^17^, ^18^, ^19^, ^20^ Information on the included research methods, objects, interventions and outcomes are shown in the following description of the included literature features (Table 1). The method of administration of hypertonic saline, the specific formulation of the solution, the treatment time and the outcome measures used in each study varied. A flowchart of study retrieval and selection is presented in Fig. 1.

**Table 1:** Characteristics of the included studies.

Authors	Year	Number of patients	Age (year)	Tonicity of HS (%)	Duration	Withdrawals	Outcomes
Shoseyov	1998	34	3–16	3.5	4 weeks	4	(1)(4)
Bachmann	2000	40	28–56	1.1	1 weeks	1	(1)(2)(3)(4)(5)
Hauptman	2007	80	21–64	3.0	10 min	0	(1)(3)(6)
Kumar	2013	50	18–45	3.5	4 weeks	8	(1)(4)
Mohan	2016	50	20–45	3.0	4 weeks	0	(1)(4)
Cai	2016	124	12–63	2.3	3 months	0	(1)(3)
Rabago	2002	76	18–65	2.0	2 weeks	7	(2)

(1), nasal symptom scores; (2), quality of life scores; (3), mucociliary clearance time; (4), radiologic scores; (5), endoscopy scores; (6), nasal reflex scores.

### Participants

**Figure 2:**
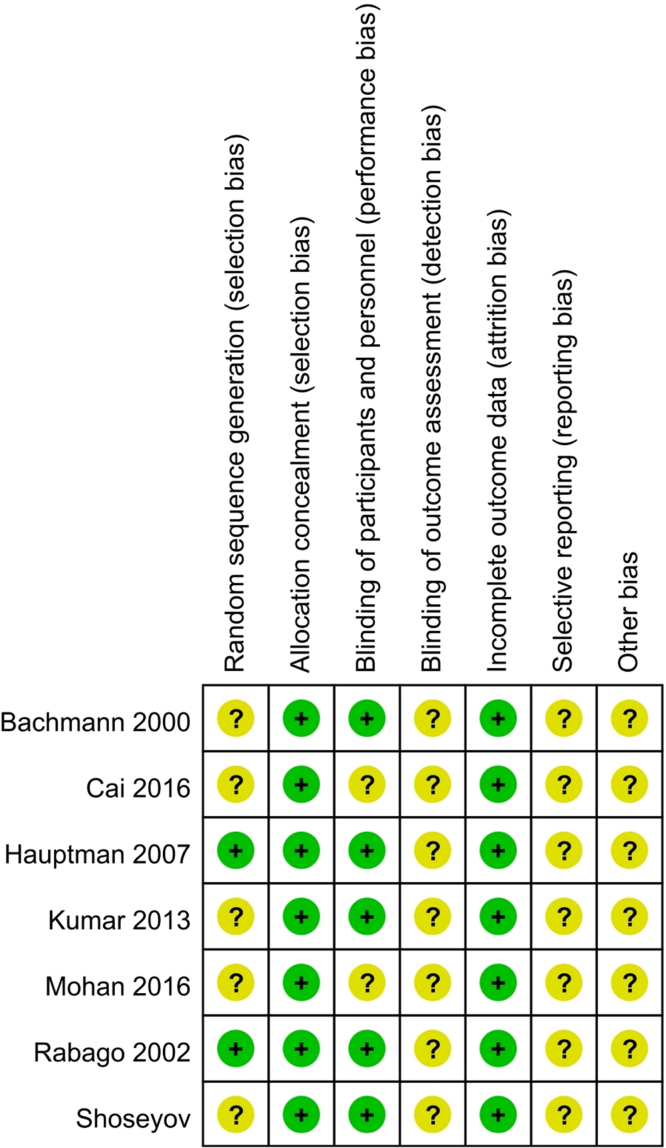
Quality of the included studies, assessed by risk of bias.

454 participants were recruited. Characteristics of the included studies are shown in Table 1. The quality of the included studies assessed according to risk of bias is shown in Fig. 2.

### Effects of interventions

#### Hypertonic saline vs. isotonic saline: nasal symptom score

**Figure 3:**
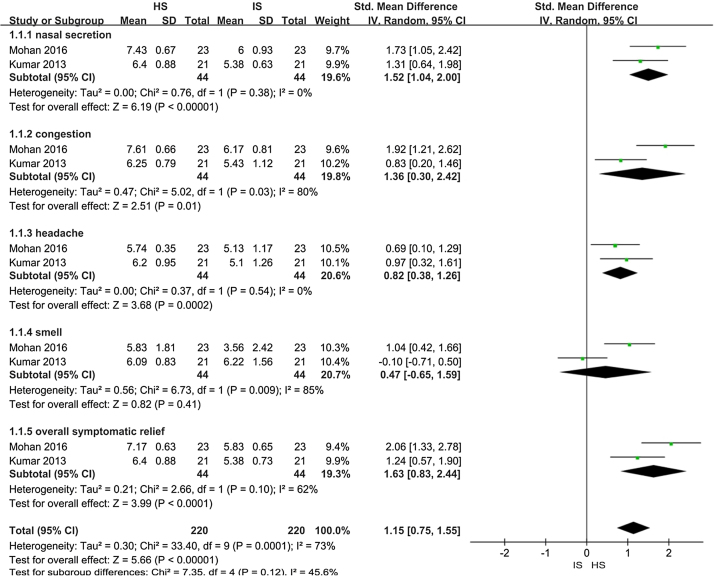
Meta-analysis of improvements in symptom scores when comparing HSNI and ISNI treatments.

Two trials assessed improvement in nasal symptom scores in patients with CRS (88 patients). When the data were pooled for meta-analysis (a forest plot is shown in Fig. 3):

1. Nasal secretion: The Hypertonic Saline Nasal Irrigation (HSNI) group showed greater improvements in the reduction of nasal secretion than the Isotonic Saline Nasal Irrigation (ISNI) group (SMD = 1.52; 95% CI: 1.04, 2.00; *p* < 0.01). There was no heterogeneity among studies (*I*^2^ = 0%).
2. Nasal congestion: The HSNI group showed greater improvements in the reduction of nasal congestion than the ISNI group (SMD = 1.36; 95% CI: 0.03, 2.42; *p* = 0.01). There was heterogeneity among studies (*I*^2^ = 80%), which may be due to the difference base between the two trails in the cardinality.
3. Headache: The HSNI group showed greater improvements in headache reduction than the ISNI group (SMD = 0.82; 95% CI: 0.38, 1.26; *p* < 0.01). There was no heterogeneity among studies (*I*^2^ = 0%).
4. Smell: There was no difference in smell between the HSNI and the ISNI group (SMD = 0.47; 95% CI: −0.65, 1.59; *p* = 0.41). The results show that it is not statistically significant.
5. Overall symptomatic relief: The HSNI group showed greater improvements in overall symptomatic relief than the ISNI group (SMD = 1.63; 95% CI: 0.83, 2.44; *p* < 0.01). There was heterogeneity among studies (*I*^2^ = 62%). It may be due to the difference base between the two trails in the cardinality.

#### Radiological scores

**Figure 4:**
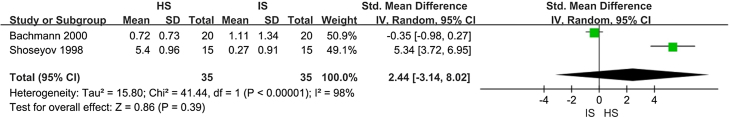
Meta-analysis of improvements in radiologic scores when comparing HSNI and ISNI treatments.

There were two trials assessing improvement in radiological scores (70 patients). When data were pooled for meta-analysis, there was no difference in imaging score between the HSNI and the ISNI groups (SMD = 2.44; 95% CI: −3.14, 8.02; *p* < 0.01). There was substantial heterogeneity (*I*^2^ = 98%). Heterogeneity may be caused by different methods of measuring results. A forest plot is shown in Fig. 4.

#### Mucociliary clearance time

**Figure 5:**
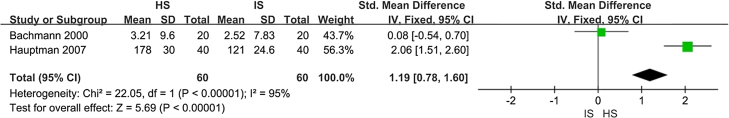
Absolute improvement in MCT when comparing HSNI and ISNI treatments.

There were two trial assessing improvement in MCT scores (120 patients). When data were pooled for meta-analysis, the HSNI group showed greater improvement in the MCT scores than did the ISNI group (SMD = 1.19; 95% CI: 0.78, 1.60; *p* < 0.01). There was substantial heterogeneity (*I*^2^ = 95%). Heterogeneity may be caused by different methods of measuring results. A forest plot is shown in Fig. 5.

#### Adverse events

**Figure 6:**
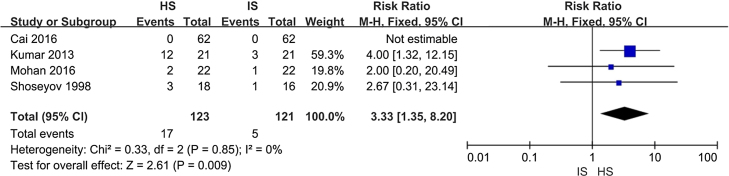
Adverse events when comparing HSNI and ISNI treatments.

As the forest plot is shown in Fig. 6, HS had higher risk (13.8%) over IS (4.1%; risk ratio 3.33; 95% CI 1.35, 8.20). Most of adverse events were nasal irritation and burning sensation. Other events included tearing, nosebleeds, headache, or nasal drainage.

## Discussion

This study disclosed significantly improved effectiveness of hypertonic saline nasal irrigation in management of CRS patients’ nasal symptoms and MCT scores, as compared to isotonic saline. However, neither hypertonic saline nor isotonic saline causes any significant improvement when evaluated by imaging. In various studies different concentrations of hypertonic saline solutions have been used. Talbot^21^ detected the mucociliary clearance rate of normal humans after nasal irrigation in buffered hypertonic saline (2%, pH = 7.6) and buffered saline groups. He found that hypertonic saline nasal irrigation can significantly improve mucociliary clearance rate. He believes that buffering hypertonic saline can increase the thickness of the mucus layer and reduce the viscosity of the mucus, which is more conducive to improved movement of the cilia. Lansley^22^ found that hypertonic saline can cause an increase in intracellular Ca^2+^ release, while Ca^2+^ increases tile rate of ciliary oscillation. They also found that hypertonic saline nasal irrigation was more effective in improving symptoms and did not increase the incidence of side effects

In terms of safety, the side effects of nasal irrigation were minimal. No uniform standard for clinical nasal irrigation exists and each person has different feelings on nasal irrigation. It is difficult to avoid certain side effects. For example, Liu^23^ has studied the effects of nasal irrigation fluid temperature on the healing time of nasal mucosa. He has found that nasal irrigation fluid of 32–34 degree centigrade does not burn or stimulate the nasal mucosa. It can also promote mucosal blood flow in the operating chamber, improve local anti-inflammatory effects and remove mucosal inflammation and edema. In addition, it is also necessary to slow adjustments according to the comfort level of the patient about pressure selection, from small to large. Different methods and different flushing fluids produce different feelings for different patients, so nasal irrigation will inevitably produce different adverse reactions in some individuals with lower tolerance.

Keeri et al. have polled 134 patients with questionnaires. They report that 95% of patients thought that nasal irrigation was a simple and easy method of treatment, and 84.7% of patients thought that this treatment was comfortable. Thus, the authors assumed that nasal irrigation is a valid treatment for patients.^24^

Some studies have examined treatment of sinus symptoms with hypertonic saline, thus further demonstrating the effectiveness of hypertonic saline in treating sinusitis. However, these studies examined not only chronic sinusitis but also acute sinusitis, allergic rhinitis and so forth. Our study involved only patients with chronic sinusitis before surgery and thus may be more valuable in guiding clinical treatment. The following limitations were identified in the present study: (1) The loss of patients was not documented in further detail, and the calculations were slightly different. (2) Some studies did not use uniform result measures. (3) Each study involved only a small sample size. (4) Various nasal irrigation methods were used. (5) The heterogeneity may be increased by comparing children with adults in evaluating CT scores. The concentrations of nasal irrigation fluid also differed. There were no strict differences between the various forms and the concentration of the rinsing solution. Future clinical randomized controlled studies should be designed to include a larger sample size. It needs to adopt more rigorous randomization methods, assign concealed and double-blinded study designs, formulate and adopt uniform therapeutic efficacy criteria, and use uniform measurement units and uniform nasal irrigation methods and rinse concentration. Also, negative results should be published. The limits above may increase the heterogeneity of the article, but it has fewer impacts on the main results of the study and has a greater impact on adverse effects.

For clinical applications, according to the results of our systematic review of the information included in the literature, it is not yet possible to recommend specific nasal irrigation methods, devices, doses or frequency. Hypertonic saline nasal irrigation produces better results than isotonic saline, and there is more evidence that hypertonic saline can better improve mucociliary clearance in patients. Different treatments should be tailored to individual patient conditions to develop a personalized washing program to gain satisfactory results. How to develop a suitable flushing program tailor-made for each patient requires a more in-depth and thorough study of nasal irrigation.

## Conclusion

Compared with isotonic saline, hypertonic saline nasal irrigation for treating chronic rhinosinusitis had mild side effects and was significantly more effective in improving nasal symptoms and ciliary movement, despite a lack of significant differences in imaging findings and smell improvement. Hypertonic saline nasal irrigation is worthy of widespread use in clinical practice, but further study of the methods and concentrations in nasal irrigation is necessary.

## Conflicts of interest

The authors declare no conflicts of interest.
